# Diethyl 4,5-diphenyl-3,6-bis­(trimethyl­sil­yl)benzene-1,2-dicarboxyl­ate

**DOI:** 10.1107/S160053681102397X

**Published:** 2011-06-30

**Authors:** Jing Zhang, Hongmei Qu, Zhenyu Zhang, Lishan Zhou

**Affiliations:** a School of Chemical Engineering and Technology, Tianjin University, Tianjin 300072, People’s Republic of China; b Tianjin Research and Design Institute of Chemical Industry, Tianjin 300131, People’s Republic of China

## Abstract

In the title compound, C_30_H_38_O_4_Si_2_, the two phenyl rings are twisted away from the central benzene ring by 70.28 (8) and 67.42 (7)°. The two Si atoms attached to the benzene ring deviate in opposite directions from the ring plane by 0.258 (3) and 0.206 (3) Å, respectively. One ethyl group is disordered over two conformations in a 0.568 (5):0.432 (5) ratio. The crystal packing exhibits weak inter­molecular C—H⋯O inter­actions.

## Related literature

For general background to the synthesis of benzene compounds, see: Reppe & Schweckendiek (1948 ▶); Reppe *et al.* (1948 ▶); Schore (1988 ▶); Vollhardt (1984 ▶); Yamamoto (2005 ▶). For related structures, see: Haberecht *et al.* (2002 ▶); Takahashi *et al.* (2006 ▶).
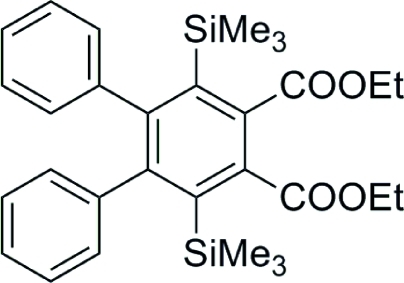


## Experimental

### 

#### Crystal data

C_30_H_38_O_4_Si_2_
*M* *_r_* = 518.78
Triclinic, 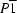

*a* = 11.534 (2) Å
*b* = 12.389 (3) Å
*c* = 12.853 (3) Å
α = 63.40 (3)°
β = 67.63 (3)°
γ = 65.99 (3)°
*V* = 1455.1 (5) Å^3^
*Z* = 2
Mo *K*α radiation
μ = 0.15 mm^−1^
*T* = 113 K
0.20 × 0.18 × 0.12 mm

#### Data collection

Rigaku Saturn CCD area-detector diffractometer
Absorption correction: multi-scan (*CrystalClear*; Rigaku/MSC, 2005 ▶) *T*_min_ = 0.970, *T*_max_ = 0.982
12066 measured reflections
6772 independent reflections
3380 reflections with *I* > 2σ(*I*)
*R*_int_ = 0.047

#### Refinement

*R*[*F* ^2^ > 2σ(*F* ^2^)] = 0.046
*wR*(*F* ^2^) = 0.127
*S* = 0.95
6772 reflections
338 parameters
4 restraints
H-atom parameters constrained
Δρ_max_ = 0.31 e Å^−3^
Δρ_min_ = −0.38 e Å^−3^

### 

Data collection: *CrystalClear* (Rigaku/MSC, 2005 ▶); cell refinement: *CrystalClear*; data reduction: *CrystalClear*; program(s) used to solve structure: *SHELXS97* (Sheldrick, 2008 ▶); program(s) used to refine structure: *SHELXL97* (Sheldrick, 2008 ▶); molecular graphics: *SHELXTL* (Sheldrick, 2008 ▶); software used to prepare material for publication: *SHELXTL*.

## Supplementary Material

Crystal structure: contains datablock(s) I, global. DOI: 10.1107/S160053681102397X/cv5108sup1.cif

Structure factors: contains datablock(s) I. DOI: 10.1107/S160053681102397X/cv5108Isup2.hkl

Supplementary material file. DOI: 10.1107/S160053681102397X/cv5108Isup3.cml

Additional supplementary materials:  crystallographic information; 3D view; checkCIF report

## Figures and Tables

**Table 1:** Hydrogen-bond geometry (Å, °)

*D*—H⋯*A*	*D*—H	H⋯*A*	*D*⋯*A*	*D*—H⋯*A*
C10—H10*B*⋯O1	0.98	2.39	3.116 (3)	131
C27—H27*B*⋯O4	0.98	2.43	3.137 (3)	129

**Fig. 1.**
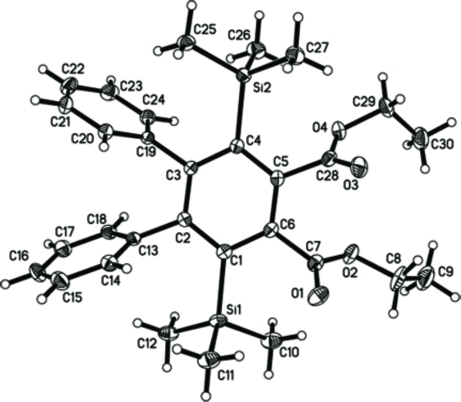
View of the molecular structure of (I). Displacement ellipsoids are drawn at the 30% probability level. Only major part of the disordered ethyl fragment is shown.

**Fig. 2.**
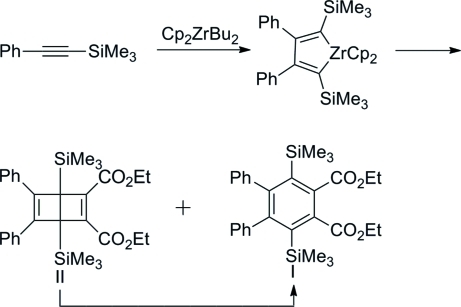
The preparation of the title compound.

## supplementary crystallographic information

### Comment

The synthesis of benzene compounds is an important activity in organic chemistry. Reppe and colleagues (Reppe & Schweckendiek, 1948; Reppe *et al.*, 1948) first discovered the Ni-catalyzed cyclization of acetylene affording benzene in 1948. Since then, Vollhardt *et al.* (1984), Schore (1988), Yamamoto (2005) and many others developed the chemistry of transition metal complexes-mediated or catalyzed cyclotrimerization of alkynes. The title compound (I) has been prepared by zirconocene-mediated cyclization of 1-phenyl-2- trimethylsilyl acetylene and diethyl acetylenedicarboxylate (DEAD). Herewith we present its crystal structure.

In (I) (Fig.1), the C1—Si1 and C4—Si2 bond lengths of 1.921 (2) and 1.923 (2) Å, respectively, are slightly longer than those in *p*-bis(trimethylsilyl)benzene [1.882 (1) Å] (Haberecht *et al.*, 2002). The lengths of the C—Si and C—O bonds in (I) agree with the corresponding values in 1,4-bis(trimethylsilyl)-2,3-bis(methoxycarbonyl)-9,10-dihydroanthracene (Takahashi *et al.*, 2006). It seems that the bond lengths are influenced by the steric hindrance of substituents on the central benzene ring. In the title molecule, there are three benzene rings -  A (C1—C6),  B (C13—C18) and  C (C19—C24), respectively. Rings B and C are twisted from the central benzene ring A at 67.42 (7) and 70.28 (8)°, respectively. The Si1 and Si2 atoms attached to benzene ring deviate from its plane in opposite directions at 0.206 (3) and 0.258 (3) Å, respectively.

The crystal packing exhibits weak intermolecular C—H···O interactions (Table 1).

### Experimental

The title compound (I) has been prepared by zirconocene-mediated cyclization of 1-phenyl-2- trimethylsilyl acetylene and diethyl acetylenedicarboxylate (DEAD) (see Fig. 2). To a solution of Cp_2_ZrCl_2_ (350 mg, 1.20 mmol) in 10 ml of THF was added *n-*BuLi (1.56 *M* hexane solution, 1.54 ml, 2.40 mmol) at -78 °C, and the mixture was stirred for 15 min. The solution was warmed to -40°C for 30 min and then re-cooled to -78°C. After 15 min, 1-phenyl-2-trimethylsilyl acetylene (393 *µ*L, 2.0 mmol) was added to the solution, and it was warmed to room temperature. After stirring for 3 h, CuCl (297 mg, 3.0 mmol) and diethyl acetylenedicarboxylate (DEAD) (0.477 ml, 3.0 mmol) were added to the mixture, and it was stirred for 6 h at room temperature. The mixture was quenched with 3 N HCl and extracted with ethyl acetate. The combined organic phase was washed with water, saturated aqueous NaHCO_3_ solution, and brine. The solution was dried over anhydrous Na_2_SO_4_. The solvent was evaporated, and the resulting solid was purified by a flash chromatography (silica gel, hexane: ethyl acetate =5:1 as eluent) to afford mixture of the title compound I and II. When mixture was heated in toluene at 100 °C for 3 h, benzene I (424 mg) was obtained in 82% yield as pale yellow solid. ^1^H NMR (CDCl3, Me4Si): -0.14 (s, 18 H), 1.41 (t, J = 6.9 Hz, 6 H), 4.29–4.36 (q, J = 7.2 Hz, 4 H), 6.78–6.81 (m, 4 H), 7.00–7.08 (m, 6 H); ^13^C NMR (CDCl3, Me4Si): 1.7, 13.8, 61.8, 126.7, 126.9, 131.3, 137.9, 138.0, 142.0, 149.6, 170.5. HRMS (EI) calc. for C_30_H_38_O_4_Si_2_: 518.2309. Found: 518.2314. The solid of compound (I) was re-crystallized by ethanol to give colorless single crystals of (I), suitable for X-ray analysis.

### Refinement

The H atoms were placed in calculated positions (C—H = 0.95–0.99 Å) and constrained to ride on their parent atoms, with C—H = 0.95, 0.99 and 0.98 Å for aromatic, methylene and methyl H atoms, respectively. All H atoms were refined as riding with *U*_iso_(H) = *xU*_eq_(C), where *x* = 1.5 for methyl H, and *x* = 1.2 for the other H atoms. The ethyl chain C8—C9 has been treated as disordered over two conformations with the occupancies refined to  0.568 (5) and 0.432 (5), respectively.

### Crystal data

**Table:**

C_30_H_38_O_4_Si_2_	*Z* = 2
*M**_r_* = 518.78	*F*(000) = 556
Triclinic, *P*1	*D*_x_ = 1.184 Mg m^−^^3^
Hall symbol: -P 1	Mo *K*α radiation, λ = 0.71073 Å
*a* = 11.534 (2) Å	Cell parameters from 4247 reflections
*b* = 12.389 (3) Å	θ = 1.9–27.9°
*c* = 12.853 (3) Å	µ = 0.15 mm^−^^1^
α = 63.40 (3)°	*T* = 113 K
β = 67.63 (3)°	Block, colourless
γ = 65.99 (3)°	0.20 × 0.18 × 0.12 mm
*V* = 1455.1 (5) Å^3^	

### Data collection

**Table:**

Rigaku Saturn CCD area-detector diffractometer	6772 independent reflections
Radiation source: rotating anode	3380 reflections with *I* > 2σ(*I*)
multilayer	*R*_int_ = 0.047
Detector resolution: 7.31 pixels mm^-1^	θ_max_ = 27.9°, θ_min_ = 1.9°
ω and φ scans	*h* = −15→12
Absorption correction: multi-scan (*CrystalClear*; Rigaku/MSC, 2005)	*k* = −15→16
*T*_min_ = 0.970, *T*_max_ = 0.982	*l* = −16→13
12066 measured reflections	

### Refinement

**Table:**

Refinement on *F*^2^	Primary atom site location: structure-invariant direct methods
Least-squares matrix: full	Secondary atom site location: difference Fourier map
*R*[*F*^2^ > 2σ(*F*^2^)] = 0.046	Hydrogen site location: inferred from neighbouring sites
*wR*(*F*^2^) = 0.127	H-atom parameters constrained
*S* = 0.95	*w* = 1/[σ^2^(*F*_o_^2^) + (0.0512*P*)^2^] where *P* = (*F*_o_^2^ + 2*F*_c_^2^)/3
6772 reflections	(Δ/σ)_max_ = 0.001
338 parameters	Δρ_max_ = 0.31 e Å^−^^3^
4 restraints	Δρ_min_ = −0.38 e Å^−^^3^

### Special details

**Table:**

Geometry. All e.s.d.'s (except the e.s.d. in the dihedral angle between two l.s. planes) are estimated using the full covariance matrix. The cell e.s.d.'s are taken into account individually in the estimation of e.s.d.'s in distances, angles and torsion angles; correlations between e.s.d.'s in cell parameters are only used when they are defined by crystal symmetry. An approximate (isotropic) treatment of cell e.s.d.'s is used for estimating e.s.d.'s involving l.s. planes.
Refinement. Refinement of *F*^2^ against ALL reflections. The weighted *R*-factor *wR* and goodness of fit *S* are based on *F*^2^, conventional *R*-factors *R* are based on *F*, with *F* set to zero for negative *F*^2^. The threshold expression of *F*^2^ > σ(*F*^2^) is used only for calculating *R*-factors(gt) *etc*. and is not relevant to the choice of reflections for refinement. *R*-factors based on *F*^2^ are statistically about twice as large as those based on *F*, and *R*- factors based on ALL data will be even larger.

### Fractional atomic coordinates and isotropic or equivalent isotropic displacement parameters (Å^2^)

**Table:**

	*x*	*y*	*z*	*U*_iso_*/*U*_eq_	Occ. (<1)
Si1	0.73346 (5)	0.73464 (5)	1.08693 (5)	0.02948 (16)	
Si2	0.27477 (5)	0.80890 (5)	0.83890 (5)	0.02739 (15)	
O1	0.77590 (13)	0.91083 (15)	0.81335 (14)	0.0447 (4)	
O2	0.81690 (12)	0.76114 (14)	0.74003 (13)	0.0418 (4)	
O3	0.59560 (14)	0.92819 (15)	0.63566 (13)	0.0451 (4)	
O4	0.56124 (12)	0.74465 (14)	0.67925 (11)	0.0349 (4)	
C1	0.59901 (16)	0.76175 (17)	1.01700 (17)	0.0245 (4)	
C2	0.47780 (16)	0.73924 (17)	1.08678 (16)	0.0232 (4)	
C3	0.38661 (16)	0.74403 (17)	1.03477 (16)	0.0216 (4)	
C4	0.40993 (16)	0.77728 (17)	0.91040 (16)	0.0231 (4)	
C5	0.53145 (17)	0.79973 (18)	0.84046 (16)	0.0247 (4)	
C6	0.62236 (16)	0.79331 (17)	0.89246 (17)	0.0252 (4)	
C7	0.74488 (18)	0.8302 (2)	0.81181 (18)	0.0312 (5)	
C8	0.9196 (6)	0.8173 (6)	0.6438 (7)	0.0543 (17)	0.568 (5)
H8A	0.9635	0.8450	0.6763	0.065*	0.568 (5)
H8B	0.8814	0.8906	0.5795	0.065*	0.568 (5)
C9	1.0145 (4)	0.7134 (5)	0.5967 (4)	0.0716 (19)	0.568 (5)
H9A	1.0914	0.7403	0.5398	0.107*	0.568 (5)
H9B	0.9722	0.6946	0.5560	0.107*	0.568 (5)
H9C	1.0417	0.6378	0.6635	0.107*	0.568 (5)
C8'	0.9492 (8)	0.7716 (10)	0.6688 (10)	0.0543 (17)	0.432 (5)
H8'A	1.0016	0.6964	0.6453	0.065*	0.432 (5)
H8'B	0.9947	0.7790	0.7159	0.065*	0.432 (5)
C9'	0.9316 (6)	0.8881 (7)	0.5594 (6)	0.090 (3)	0.432 (5)
H9'A	1.0171	0.9018	0.5115	0.136*	0.432 (5)
H9'B	0.8752	0.9609	0.5841	0.136*	0.432 (5)
H9'C	0.8912	0.8774	0.5113	0.136*	0.432 (5)
C10	0.89613 (18)	0.6606 (2)	1.0013 (2)	0.0452 (6)	
H10A	0.9650	0.6464	1.0368	0.068*	
H10B	0.9123	0.7168	0.9175	0.068*	
H10C	0.8962	0.5799	1.0047	0.068*	
C11	0.7302 (2)	0.8833 (2)	1.0921 (2)	0.0440 (6)	
H11A	0.7997	0.8663	1.1282	0.066*	
H11B	0.6452	0.9175	1.1403	0.066*	
H11C	0.7441	0.9446	1.0105	0.066*	
C12	0.72081 (19)	0.6150 (2)	1.24164 (19)	0.0405 (6)	
H12A	0.7912	0.6043	1.2734	0.061*	
H12B	0.7286	0.5344	1.2391	0.061*	
H12C	0.6360	0.6435	1.2936	0.061*	
C13	0.43822 (16)	0.71655 (18)	1.21850 (16)	0.0248 (4)	
C14	0.41489 (17)	0.8136 (2)	1.25575 (18)	0.0318 (5)	
H14A	0.4273	0.8919	1.1977	0.038*	
C15	0.3737 (2)	0.7989 (2)	1.3765 (2)	0.0438 (6)	
H15A	0.3586	0.8665	1.4008	0.053*	
C16	0.3547 (2)	0.6856 (2)	1.4612 (2)	0.0464 (6)	
H16A	0.3266	0.6748	1.5441	0.056*	
C17	0.37663 (19)	0.5881 (2)	1.42545 (19)	0.0414 (6)	
H17A	0.3634	0.5102	1.4839	0.050*	
C18	0.41791 (17)	0.60276 (19)	1.30465 (17)	0.0302 (5)	
H18A	0.4323	0.5352	1.2807	0.036*	
C19	0.26591 (16)	0.70700 (19)	1.11783 (16)	0.0242 (4)	
C20	0.16765 (17)	0.7812 (2)	1.18300 (17)	0.0322 (5)	
H20A	0.1745	0.8595	1.1726	0.039*	
C21	0.05982 (19)	0.7405 (2)	1.26302 (19)	0.0436 (6)	
H21A	−0.0072	0.7912	1.3070	0.052*	
C22	0.0498 (2)	0.6273 (3)	1.2788 (2)	0.0483 (7)	
H22A	−0.0237	0.5996	1.3342	0.058*	
C23	0.1457 (2)	0.5537 (2)	1.21452 (19)	0.0434 (6)	
H23A	0.1383	0.4756	1.2250	0.052*	
C24	0.25341 (18)	0.5944 (2)	1.13433 (18)	0.0324 (5)	
H24A	0.3196	0.5434	1.0901	0.039*	
C25	0.10646 (17)	0.8685 (2)	0.92639 (18)	0.0355 (5)	
H25A	0.0419	0.8829	0.8859	0.053*	
H25B	0.0982	0.9477	0.9320	0.053*	
H25C	0.0910	0.8060	1.0072	0.053*	
C26	0.2847 (2)	0.6650 (2)	0.8199 (2)	0.0428 (6)	
H26A	0.2147	0.6837	0.7835	0.064*	
H26B	0.2747	0.5979	0.8984	0.064*	
H26C	0.3697	0.6375	0.7677	0.064*	
C27	0.28319 (19)	0.9412 (2)	0.69133 (18)	0.0433 (6)	
H27A	0.2133	0.9556	0.6570	0.065*	
H27B	0.3683	0.9194	0.6365	0.065*	
H27C	0.2725	1.0179	0.7032	0.065*	
C28	0.56678 (17)	0.8339 (2)	0.70744 (18)	0.0308 (5)	
C29	0.5882 (2)	0.7649 (2)	0.55243 (18)	0.0455 (6)	
H29A	0.5612	0.8563	0.5071	0.055*	
H29B	0.5362	0.7248	0.5424	0.055*	
C30	0.7289 (2)	0.7116 (2)	0.5031 (2)	0.0568 (7)	
H30A	0.7442	0.7265	0.4180	0.085*	
H30B	0.7554	0.6207	0.5469	0.085*	
H30C	0.7804	0.7521	0.5118	0.085*	

### Atomic displacement parameters (Å^2^)

**Table:**

	*U*^11^	*U*^22^	*U*^33^	*U*^12^	*U*^13^	*U*^23^
Si1	0.0278 (3)	0.0246 (3)	0.0402 (3)	−0.0068 (2)	−0.0132 (2)	−0.0112 (3)
Si2	0.0273 (3)	0.0293 (4)	0.0268 (3)	−0.0070 (2)	−0.0075 (2)	−0.0109 (2)
O1	0.0424 (8)	0.0370 (10)	0.0554 (10)	−0.0222 (7)	−0.0068 (7)	−0.0114 (8)
O2	0.0315 (8)	0.0469 (11)	0.0420 (9)	−0.0185 (7)	0.0080 (7)	−0.0186 (8)
O3	0.0552 (9)	0.0424 (10)	0.0303 (8)	−0.0258 (8)	−0.0077 (7)	0.0019 (7)
O4	0.0415 (8)	0.0350 (9)	0.0242 (7)	−0.0102 (7)	−0.0028 (6)	−0.0119 (7)
C1	0.0256 (10)	0.0159 (10)	0.0307 (11)	−0.0042 (8)	−0.0075 (8)	−0.0080 (8)
C2	0.0259 (10)	0.0177 (10)	0.0243 (10)	−0.0036 (8)	−0.0071 (8)	−0.0073 (8)
C3	0.0211 (9)	0.0173 (11)	0.0250 (10)	−0.0045 (8)	−0.0045 (8)	−0.0080 (8)
C4	0.0229 (9)	0.0189 (11)	0.0252 (10)	−0.0044 (8)	−0.0047 (8)	−0.0082 (8)
C5	0.0270 (10)	0.0194 (11)	0.0232 (10)	−0.0051 (8)	−0.0043 (8)	−0.0065 (8)
C6	0.0231 (10)	0.0189 (11)	0.0291 (11)	−0.0058 (8)	−0.0049 (8)	−0.0059 (8)
C7	0.0288 (11)	0.0266 (13)	0.0319 (12)	−0.0082 (9)	−0.0069 (9)	−0.0050 (10)
C8	0.036 (3)	0.051 (5)	0.051 (4)	−0.022 (3)	0.015 (3)	−0.009 (4)
C9	0.042 (3)	0.092 (5)	0.062 (3)	−0.024 (3)	0.011 (2)	−0.027 (3)
C8'	0.036 (3)	0.051 (5)	0.051 (4)	−0.022 (3)	0.015 (3)	−0.009 (4)
C9'	0.061 (4)	0.126 (8)	0.049 (4)	−0.049 (5)	0.008 (4)	0.002 (5)
C10	0.0333 (12)	0.0412 (15)	0.0637 (16)	−0.0005 (10)	−0.0194 (11)	−0.0232 (13)
C11	0.0426 (13)	0.0364 (14)	0.0635 (16)	−0.0133 (11)	−0.0155 (11)	−0.0221 (12)
C12	0.0398 (12)	0.0375 (14)	0.0481 (14)	−0.0075 (10)	−0.0256 (10)	−0.0089 (11)
C13	0.0217 (10)	0.0253 (12)	0.0266 (10)	−0.0042 (8)	−0.0082 (8)	−0.0087 (9)
C14	0.0323 (11)	0.0293 (13)	0.0334 (12)	−0.0057 (9)	−0.0107 (9)	−0.0111 (10)
C15	0.0460 (13)	0.0479 (16)	0.0445 (14)	−0.0051 (11)	−0.0153 (11)	−0.0259 (12)
C16	0.0520 (14)	0.0612 (19)	0.0263 (12)	−0.0136 (13)	−0.0138 (10)	−0.0144 (12)
C17	0.0440 (13)	0.0455 (16)	0.0294 (12)	−0.0165 (11)	−0.0144 (10)	−0.0007 (11)
C18	0.0329 (11)	0.0289 (12)	0.0291 (11)	−0.0093 (9)	−0.0126 (9)	−0.0056 (9)
C19	0.0216 (9)	0.0282 (12)	0.0211 (10)	−0.0071 (8)	−0.0057 (8)	−0.0066 (8)
C20	0.0283 (11)	0.0378 (14)	0.0294 (11)	−0.0090 (9)	−0.0053 (9)	−0.0125 (10)
C21	0.0281 (12)	0.0634 (19)	0.0317 (12)	−0.0086 (11)	0.0003 (9)	−0.0202 (12)
C22	0.0343 (13)	0.0666 (19)	0.0389 (14)	−0.0277 (13)	−0.0005 (10)	−0.0091 (13)
C23	0.0456 (13)	0.0450 (16)	0.0432 (14)	−0.0268 (12)	−0.0116 (11)	−0.0053 (12)
C24	0.0330 (11)	0.0337 (13)	0.0297 (11)	−0.0127 (9)	−0.0076 (9)	−0.0076 (10)
C25	0.0295 (11)	0.0367 (14)	0.0400 (12)	−0.0044 (9)	−0.0123 (9)	−0.0144 (10)
C26	0.0477 (13)	0.0431 (15)	0.0496 (14)	−0.0125 (11)	−0.0132 (11)	−0.0252 (12)
C27	0.0389 (12)	0.0437 (15)	0.0352 (12)	−0.0032 (10)	−0.0155 (10)	−0.0059 (11)
C28	0.0236 (10)	0.0311 (13)	0.0291 (11)	−0.0073 (9)	−0.0053 (8)	−0.0048 (10)
C29	0.0528 (14)	0.0563 (17)	0.0248 (11)	−0.0159 (12)	−0.0067 (10)	−0.0138 (11)
C30	0.0553 (15)	0.070 (2)	0.0390 (14)	−0.0144 (13)	0.0011 (12)	−0.0271 (14)

### Geometric parameters (Å, °)

**Table:**

Si1—C11	1.858 (2)	C11—H11C	0.9800
Si1—C12	1.868 (2)	C12—H12A	0.9800
Si1—C10	1.872 (2)	C12—H12B	0.9800
Si1—C1	1.9214 (19)	C12—H12C	0.9800
Si2—C26	1.859 (2)	C13—C14	1.379 (3)
Si2—C25	1.869 (2)	C13—C18	1.394 (3)
Si2—C27	1.871 (2)	C14—C15	1.387 (3)
Si2—C4	1.9233 (19)	C14—H14A	0.9500
O1—C7	1.200 (2)	C15—C16	1.380 (3)
O2—C7	1.348 (2)	C15—H15A	0.9500
O2—C8'	1.475 (8)	C16—C17	1.376 (3)
O2—C8	1.483 (6)	C16—H16A	0.9500
O3—C28	1.202 (2)	C17—C18	1.387 (3)
O4—C28	1.338 (2)	C17—H17A	0.9500
O4—C29	1.459 (2)	C18—H18A	0.9500
C1—C2	1.406 (2)	C19—C24	1.374 (3)
C1—C6	1.409 (3)	C19—C20	1.397 (3)
C2—C3	1.416 (2)	C20—C21	1.390 (3)
C2—C13	1.498 (2)	C20—H20A	0.9500
C3—C4	1.404 (2)	C21—C22	1.372 (3)
C3—C19	1.496 (2)	C21—H21A	0.9500
C4—C5	1.409 (2)	C22—C23	1.377 (3)
C5—C6	1.404 (3)	C22—H22A	0.9500
C5—C28	1.493 (3)	C23—C24	1.389 (3)
C6—C7	1.505 (3)	C23—H23A	0.9500
C8—C9	1.506 (8)	C24—H24A	0.9500
C8—H8A	0.9900	C25—H25A	0.9800
C8—H8B	0.9900	C25—H25B	0.9800
C9—H9A	0.9800	C25—H25C	0.9800
C9—H9B	0.9800	C26—H26A	0.9800
C9—H9C	0.9800	C26—H26B	0.9800
C8'—C9'	1.504 (8)	C26—H26C	0.9800
C8'—H8'A	0.9900	C27—H27A	0.9800
C8'—H8'B	0.9900	C27—H27B	0.9800
C9'—H9'A	0.9800	C27—H27C	0.9800
C9'—H9'B	0.9800	C29—C30	1.481 (3)
C9'—H9'C	0.9800	C29—H29A	0.9900
C10—H10A	0.9800	C29—H29B	0.9900
C10—H10B	0.9800	C30—H30A	0.9800
C10—H10C	0.9800	C30—H30B	0.9800
C11—H11A	0.9800	C30—H30C	0.9800
C11—H11B	0.9800		
			
C11—Si1—C12	108.75 (11)	Si1—C12—H12B	109.5
C11—Si1—C10	111.67 (10)	H12A—C12—H12B	109.5
C12—Si1—C10	102.99 (11)	Si1—C12—H12C	109.5
C11—Si1—C1	111.51 (9)	H12A—C12—H12C	109.5
C12—Si1—C1	112.47 (9)	H12B—C12—H12C	109.5
C10—Si1—C1	109.18 (9)	C14—C13—C18	118.75 (19)
C26—Si2—C25	108.78 (10)	C14—C13—C2	118.88 (18)
C26—Si2—C27	110.16 (11)	C18—C13—C2	122.30 (19)
C25—Si2—C27	102.15 (10)	C13—C14—C15	121.2 (2)
C26—Si2—C4	111.22 (9)	C13—C14—H14A	119.4
C25—Si2—C4	113.09 (8)	C15—C14—H14A	119.4
C27—Si2—C4	111.07 (10)	C16—C15—C14	119.6 (2)
C7—O2—C8'	119.7 (7)	C16—C15—H15A	120.2
C7—O2—C8	111.5 (4)	C14—C15—H15A	120.2
C8'—O2—C8	21.5 (4)	C17—C16—C15	119.9 (2)
C28—O4—C29	117.80 (17)	C17—C16—H16A	120.1
C2—C1—C6	116.18 (16)	C15—C16—H16A	120.1
C2—C1—Si1	122.45 (14)	C16—C17—C18	120.5 (2)
C6—C1—Si1	121.06 (13)	C16—C17—H17A	119.8
C1—C2—C3	121.63 (16)	C18—C17—H17A	119.8
C1—C2—C13	120.41 (15)	C17—C18—C13	120.1 (2)
C3—C2—C13	117.86 (15)	C17—C18—H18A	120.0
C4—C3—C2	121.75 (16)	C13—C18—H18A	120.0
C4—C3—C19	120.70 (15)	C24—C19—C20	118.84 (18)
C2—C3—C19	117.50 (15)	C24—C19—C3	119.20 (17)
C3—C4—C5	116.57 (15)	C20—C19—C3	121.90 (18)
C3—C4—Si2	121.16 (13)	C21—C20—C19	119.9 (2)
C5—C4—Si2	121.90 (13)	C21—C20—H20A	120.0
C6—C5—C4	121.50 (16)	C19—C20—H20A	120.0
C6—C5—C28	117.65 (16)	C22—C21—C20	120.3 (2)
C4—C5—C28	120.84 (16)	C22—C21—H21A	119.9
C5—C6—C1	122.30 (16)	C20—C21—H21A	119.9
C5—C6—C7	118.89 (16)	C21—C22—C23	120.3 (2)
C1—C6—C7	118.67 (16)	C21—C22—H22A	119.9
O1—C7—O2	123.88 (18)	C23—C22—H22A	119.9
O1—C7—C6	123.30 (19)	C22—C23—C24	119.5 (2)
O2—C7—C6	112.80 (18)	C22—C23—H23A	120.3
O2—C8—C9	105.0 (5)	C24—C23—H23A	120.3
O2—C8—H8A	110.8	C19—C24—C23	121.2 (2)
C9—C8—H8A	110.8	C19—C24—H24A	119.4
O2—C8—H8B	110.8	C23—C24—H24A	119.4
C9—C8—H8B	110.8	Si2—C25—H25A	109.5
H8A—C8—H8B	108.8	Si2—C25—H25B	109.5
C8—C9—H9A	109.5	H25A—C25—H25B	109.5
C8—C9—H9B	109.5	Si2—C25—H25C	109.5
H9A—C9—H9B	109.5	H25A—C25—H25C	109.5
C8—C9—H9C	109.5	H25B—C25—H25C	109.5
H9A—C9—H9C	109.5	Si2—C26—H26A	109.5
H9B—C9—H9C	109.5	Si2—C26—H26B	109.5
O2—C8'—C9'	106.5 (6)	H26A—C26—H26B	109.5
O2—C8'—H8'A	110.4	Si2—C26—H26C	109.5
C9'—C8'—H8'A	110.4	H26A—C26—H26C	109.5
O2—C8'—H8'B	110.4	H26B—C26—H26C	109.5
C9'—C8'—H8'B	110.4	Si2—C27—H27A	109.5
H8'A—C8'—H8'B	108.6	Si2—C27—H27B	109.5
C8'—C9'—H9'A	109.5	H27A—C27—H27B	109.5
C8'—C9'—H9'B	109.5	Si2—C27—H27C	109.5
H9'A—C9'—H9'B	109.5	H27A—C27—H27C	109.5
C8'—C9'—H9'C	109.5	H27B—C27—H27C	109.5
H9'A—C9'—H9'C	109.5	O3—C28—O4	124.7 (2)
H9'B—C9'—H9'C	109.5	O3—C28—C5	125.0 (2)
Si1—C10—H10A	109.5	O4—C28—C5	110.34 (17)
Si1—C10—H10B	109.5	O4—C29—C30	111.25 (17)
H10A—C10—H10B	109.5	O4—C29—H29A	109.4
Si1—C10—H10C	109.5	C30—C29—H29A	109.4
H10A—C10—H10C	109.5	O4—C29—H29B	109.4
H10B—C10—H10C	109.5	C30—C29—H29B	109.4
Si1—C11—H11A	109.5	H29A—C29—H29B	108.0
Si1—C11—H11B	109.5	C29—C30—H30A	109.5
H11A—C11—H11B	109.5	C29—C30—H30B	109.5
Si1—C11—H11C	109.5	H30A—C30—H30B	109.5
H11A—C11—H11C	109.5	C29—C30—H30C	109.5
H11B—C11—H11C	109.5	H30A—C30—H30C	109.5
Si1—C12—H12A	109.5	H30B—C30—H30C	109.5
			
C11—Si1—C1—C2	−96.97 (18)	C5—C6—C7—O1	123.1 (2)
C12—Si1—C1—C2	25.50 (19)	C1—C6—C7—O1	−52.7 (3)
C10—Si1—C1—C2	139.16 (16)	C5—C6—C7—O2	−58.3 (2)
C11—Si1—C1—C6	89.57 (18)	C1—C6—C7—O2	125.97 (19)
C12—Si1—C1—C6	−147.96 (16)	C7—O2—C8—C9	163.3 (4)
C10—Si1—C1—C6	−34.30 (19)	C8'—O2—C8—C9	46 (2)
C6—C1—C2—C3	2.0 (3)	C7—O2—C8'—C9'	−81.0 (11)
Si1—C1—C2—C3	−171.72 (14)	C8—O2—C8'—C9'	−8(2)
C6—C1—C2—C13	−174.44 (17)	C1—C2—C13—C14	66.6 (2)
Si1—C1—C2—C13	11.8 (2)	C3—C2—C13—C14	−110.0 (2)
C1—C2—C3—C4	−3.1 (3)	C1—C2—C13—C18	−116.6 (2)
C13—C2—C3—C4	173.50 (17)	C3—C2—C13—C18	66.8 (2)
C1—C2—C3—C19	174.46 (17)	C18—C13—C14—C15	0.9 (3)
C13—C2—C3—C19	−9.0 (2)	C2—C13—C14—C15	177.77 (16)
C2—C3—C4—C5	3.0 (3)	C13—C14—C15—C16	−0.4 (3)
C19—C3—C4—C5	−174.47 (16)	C14—C15—C16—C17	−0.1 (3)
C2—C3—C4—Si2	−170.20 (14)	C15—C16—C17—C18	0.1 (3)
C19—C3—C4—Si2	12.4 (2)	C16—C17—C18—C13	0.4 (3)
C26—Si2—C4—C3	−94.38 (17)	C14—C13—C18—C17	−0.8 (3)
C25—Si2—C4—C3	28.36 (19)	C2—C13—C18—C17	−177.62 (15)
C27—Si2—C4—C3	142.54 (16)	C4—C3—C19—C24	69.9 (2)
C26—Si2—C4—C5	92.81 (18)	C2—C3—C19—C24	−107.7 (2)
C25—Si2—C4—C5	−144.45 (16)	C4—C3—C19—C20	−112.8 (2)
C27—Si2—C4—C5	−30.27 (18)	C2—C3—C19—C20	69.7 (2)
C3—C4—C5—C6	−2.1 (3)	C24—C19—C20—C21	0.1 (3)
Si2—C4—C5—C6	171.00 (14)	C3—C19—C20—C21	−177.23 (16)
C3—C4—C5—C28	179.11 (17)	C19—C20—C21—C22	0.3 (3)
Si2—C4—C5—C28	−7.8 (3)	C20—C21—C22—C23	−0.7 (3)
C4—C5—C6—C1	1.3 (3)	C21—C22—C23—C24	0.6 (3)
C28—C5—C6—C1	−179.87 (18)	C20—C19—C24—C23	−0.3 (3)
C4—C5—C6—C7	−174.30 (17)	C3—C19—C24—C23	177.18 (17)
C28—C5—C6—C7	4.5 (3)	C22—C23—C24—C19	−0.1 (3)
C2—C1—C6—C5	−1.2 (3)	C29—O4—C28—O3	−2.0 (3)
Si1—C1—C6—C5	172.65 (14)	C29—O4—C28—C5	177.76 (14)
C2—C1—C6—C7	174.43 (17)	C6—C5—C28—O3	−56.6 (3)
Si1—C1—C6—C7	−11.7 (2)	C4—C5—C28—O3	122.3 (2)
C8'—O2—C7—O1	7.8 (5)	C6—C5—C28—O4	123.73 (18)
C8—O2—C7—O1	−14.2 (4)	C4—C5—C28—O4	−57.5 (2)
C8'—O2—C7—C6	−170.8 (4)	C28—O4—C29—C30	90.7 (2)
C8—O2—C7—C6	167.2 (4)		

### Hydrogen-bond geometry (Å, °)

**Table:**

*D*—H···*A*	*D*—H	H···*A*	*D*···*A*	*D*—H···*A*
C10—H10B···O1	0.98	2.39	3.116 (3)	131
C27—H27B···O4	0.98	2.43	3.137 (3)	129
